# A Storable Mediatorless Electrochemical Biosensor for Herbicide Detection

**DOI:** 10.3390/microorganisms7120630

**Published:** 2019-11-29

**Authors:** Matteo Tucci, Paolo Bombelli, Christopher J. Howe, Silvia Vignolini, Stefano Bocchi, Andrea Schievano

**Affiliations:** 1 e-Bio Center, Department of Environmental Science and Policy, Università degli Studi di Milano, via Celoria 2, 20,133 Milan, Italy; matteo.tucci@unimi.it (M.T.); andrea.schievano@unimi.it (A.S.); 2 Dipartimento di Scienze e Politiche Ambientali, Università degli Studi di Milano, Via Celoria, 2, 20,133 Milano, Italy; stefano.bocchi@unimi.it; 3 Department of Biochemistry, University of Cambridge, Hopkins Building, Downing Site, Tennis Court Road, Cambridge CB2 1QW, UK; ch26@cam.ac.uk; 4 Department of Chemistry, University of Cambridge, Lensfield Road, Cambridge CB2 1EW, UK; sv319@cam.ac.uk

**Keywords:** mediatorless, amperometric biosensor, herbicide, atrazine, diuron, paraquat, photocurrent inhibition

## Abstract

A novel mediatorless photo-bioelectrochemical sensor operated with a biofilm of the cyanobacterium *Synechocystis* PCC6803 *wt*. for herbicide detection with long term stability (>20 days) was successfully developed and tested. Photoanodic current generation was obtained in the absence of artificial mediators. The inhibitory effect on photocurrent of three commonly used herbicides (i.e., atrazine, diuron, and paraquat) was used as a means of measuring their concentrations in aqueous solution. The injection of atrazine and diuron into the algal medium caused an immediate photocurrent drop due to the inhibition of photosynthetic electron transport. The detected concentrations were suitable for environmental analysis, as revealed by a comparison with the freshwater quality benchmarks set by the Environmental Protection Agency of the United States (US EPA). In contrast, paraquat caused an initial increase (~2 h) of the photocurrent effect of about 200%, as this compound can act as a redox mediator between the cells and the anode. A relatively long-term stability of the biosensor was demonstrated, by keeping anodes colonized with cyanobacterial biofilm in the dark at 4 °C. After 22 days of storage, the performance in terms of the photocurrent was comparable with the freshly prepared biosensor. This result was confirmed by the measurement of chlorophyll content, which demonstrated preservation of the cyanobacterial biofilm. The capacity of this biosensor to recover after a cold season or other prolonged environmental stresses could be a key advantage in field applications, such as in water bodies and agriculture. This study is a step forward in the biotechnological development and implementation of storable mediatorless electrochemical biosensors for herbicide detection.

## 1. Introduction

**Figure 1:**
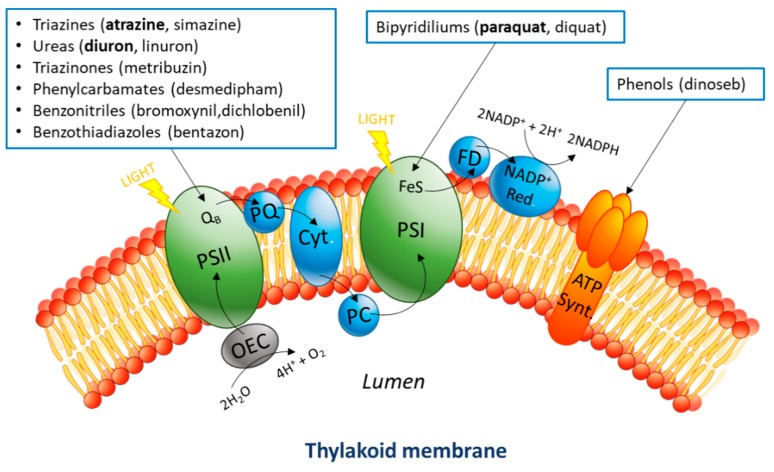
Schematic representation of the photosynthetic transport chain in the thylakoid membrane. Water oxidation happens inside the lumen and is part of a cascade of redox reactions between the different protein complexes and intermediates present in the membrane. Depending on their chemical properties, different photosynthesis inhibitors target different active sites, causing interruption of electron flow (photosystem I (PSI) and PSII) or blocking the production of ATP (ATP synthase).

Several synthetic herbicides interfere with the photosynthetic electron transport chain of photosynthetic organisms (cyanobacteria, algae, plants, etc.). Agrochemical companies have developed a variety of herbicides that exploit this to control weeds [1]. Most photosynthetic inhibitors can be categorized into one of two groups, those that inhibit photosystem II (PSII) and those that inhibit photosystem I (PSI, Figure 1).

Roughly a third of all the sales in the herbicide market are PSII inhibitors [2]. These compounds are able to block the plastoquinone binding site of PSII, thus precluding electron transfer to this intermediate [3]. Two common examples in this category are atrazine and diuron. Atrazine is a triazine herbicide widely used in sugarcane and maize agriculture [4]. Because of its persistence and extensive application, it tends to accumulate in surface and groundwater [5]. The chemical can act as an endocrine disruptor, interfering with the central nervous system and the immune system of animals and humans [6]. For these reasons, the European Union banned atrazine in 2003 [7], but it is still in use in many other countries, such as USA, Australia, and China. Diuron is another herbicide commonly used for weed control in the culture of cotton, coffee, sugar cane, and citrus [8]. Because of its widespread application, it is ubiquitous in the environment and causes serious risks to humans and animals. Moreover, its main biodegradation product, 3,4-dichloroaniline, is persistent in soil, water, and groundwater and has a higher toxicity [9].

Photosystem I inhibitors can accept electrons from PSI to form radicals, which are extremely dangerous for living cells because of their high reactivity [10]. Among them, paraquat (i.e., methyl viologen dichloride) is probably the most used, despite its high level of acute toxicity to humans and animals [11], which has led to its use even for suicide and murder [12]. In the environment, it can remain strongly adsorbed to soil particles, with a half-life up to 20 years [13]. As a consequence, this chemical was banned in the European Union [14] and several other nations. Unfortunately, it is still in use in many countries worldwide.

Given this situation, constant monitoring of these compounds in environmental compartments is crucial to ensure ecosystem and human health. Traditional analytical methods, such as high-performance liquid chromatography (HPLC), atomic absorption spectroscopy (AAS), capillary electrophoresis (CE), and mass spectrometry (MS) usually show good accuracy and precision. However, these techniques present several operational and economic limitations to their use for widespread field monitoring such as high complexity, time-consuming procedures, and requirement for sample pretreatment, expensive instrumentation, and highly trained operators [15].

To overcome these issues, researchers have focused on the development of various kinds of biosensors [16,17], employing biological elements such as antibodies [18,19,20], aptamers [21,22], and enzymes [23,24,25]. However, these kinds of bioreceptors usually require complex purification and immobilization techniques that significantly increase the overall cost of the device [26]. A novel approach to herbicide detection is represented by low-cost biosensors which are based on the cytotoxic effects of the target herbicide on photoautotrophic microorganisms. A common approach is represented by optical biosensors, where the population’s growth inhibition is analyzed by means of a spectrophotometer over time [27]. This method was found effective for detecting several different herbicides, but requires a relatively long time for cell culture and growth analysis [28]. Conversely, amperometric photobiosensors have the advantage of performing real-time monitoring of the process of inhibition [28]. This kind of sensor relies on measurement of the electron flow dependent on the bio-photocatalytic oxidation of water during the photosynthetic process. Two main types of biological materials have been employed in these systems, namely, subcellular fractions and whole cells. Examples of the first type are thylakoid membranes [29,30], photosystems [31], and reaction centers [32]. On the one hand, these have the advantages of being highly sensitive, having fast response times, and low detection limits [33]. However, on the other hand, they need complex and expensive purification techniques, are fragile after isolation, and have no self-repairing capabilities. For this reason, they seem less suitable for long-term field operations [26]. Whole cells, instead, are robust, abundant and inexpensive to culture [34,35]. Until now, amperometric biosensors based on whole cells have mainly relied on the detection of photosynthetically-produced oxygen [36,37,38,39]. However, this is an indirect measure of the photocurrent, as it can be strongly influenced by environmental factors (e.g., temperature), making the biosensors ineffective for field applications. In addition, many biosensors of this type employ selective membranes [36,37,39,40], which over long application periods can be affected by fouling.

Recently, a novel whole cell amperometric biosensor based on direct photocurrent monitoring was developed [41]. However, despite the good performance achieved, it relied on p-benzoquinone to shuttle the electrons from the photosystems inside the cells to the electrode. Quinone-based compounds are frequently used as a redox mediator in photo-bioelectrochemical devices [33,42,43,44]. They have similar chemical properties to plastoquinone, which is the natural electron acceptor in the photosynthetic electron transport chain [41]. Such compounds only temporarily increase the biofilm’s photoresponse [35], as they also typically damage the biological structures [45]. Indeed, quinones are known to be good Michael acceptors and they are able to react with macromolecules such as proteins, lipids, and DNA, as well as disrupt them [46]. In order to build long-lasting biosensors and to avoid genotoxic and cytotoxic effects caused by these chemicals, artificial mediators such as these should be avoided. Extracellular electron transfer (EET) in the absence of artificial redox mediators has been successfully demonstrated in the field of biophotovoltaics (BPVs), where photo-bioelectrochemical devices are used to produce electric power [35,47,48,49].

In this study, we designed and fabricated a novel photo-bioelectrochemical sensor for herbicide detection, operating in the absence of any artificial electron mediators. The cyanobacterium *Synechocystis* PCC6803 (wild type) was used as the photoactive component because of its proven electrogenic operation in BPV devices. [35,47,48,49,50]. A novel anodic material made of filter paper coated with carbon nanotubes and a titanium nanolayer was used. Living cyanobacterial cells were allowed to form a spontaneous biofilm on the surface of a carbon/titanium electrode. The biosensor photoresponse for diuron, atrazine, and paraquat was tested and compared. In addition, the stability of the biosensor was analyzed to assess its durability under storage conditions.

## 2. Materials and Methods

### 2.1. Reagents and Solutions

All reagents were purchased from Sigma-Aldrich (Saint Louis, MO, USA). The stock solutions of the herbicides were prepared as follows: atrazine (0.28 mM) in ethanol and water (ratio 1:7), diuron in pure ethanol (0.09 mM), and paraquat in ultrapure water (0.23 mM). Ultrapure water purified with a Millipore Milli-Q^®^ Integral purification system (Merck, Darmstadt, Germany) was used for the stock solutions. Distilled water was used for the bacterial medium. 

### 2.2. Synechocystis Culture

Wild type *Synechocystis* PCC6803 was obtained from laboratory stocks. It was cultured at room temperature (22 ± 2 °C) in a 1.5 L flask using BG11 liquid medium [51]. The source of illumination was natural sunlight. Bubbling with sterile filtered air was continuously performed in the culture to provide stirring and facilitate gas exchange. The culture was maintained at a stationary phase by removing 10% of the volume and replacing it with fresh medium every week. The optical density of the culture at 750 nm (OD750), measured with a UV-Vis spectrometer Agilent Cary 4000 (Agilent, Santa Clara, CA, USA), was maintained at 7 ± 2.

### 2.3. Electrode Fabrication and Biosensor Construction

To build the electrode, a filter paper sheet was covered with 7 layers of single walled carbon nanotube paint (SWCNT ink, Sigma-Aldrich). After each layer, the paint was allowed to dry for 1 h. The weight of nanotubes deposited for each layer was determined to be 1.12 ± 0.05 µg cm^−2^. After that, a titanium nanolayer was deposited on the surface of the electrode by evaporation, using a four-crucible e-beam evaporator Kurt J Lesker PVD 75 (KJLC, Jefferson Hills, PA, USA). To prepare the biosensor, the electrode was placed in a Petri dish and submerged in the cyanobacterial culture. The microbial cells were allowed to settle spontaneously on the surface under gravity for 48 h. Finally, the bioelectrode was dried for 15 min before electrochemical analysis to assist the physical adsorption of the cells onto the electrode surface.

### 2.4. Electrochemical Analysis

**Figure 2:**
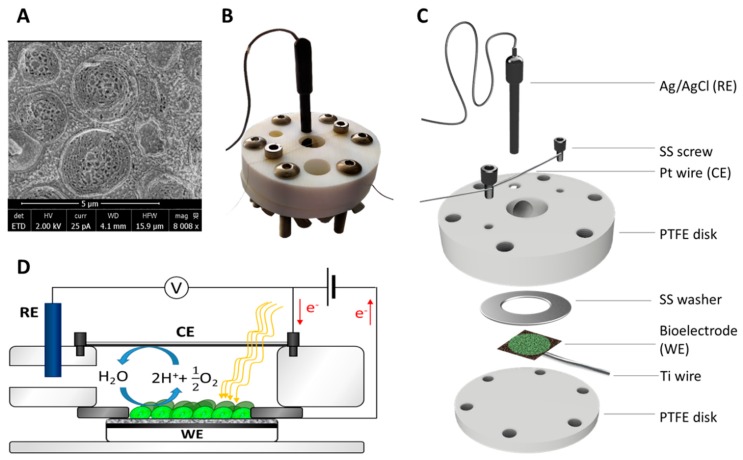
(**A**) Cryo-SEM image of Synechocystis cells, (**B**) picture, (**C**) semi-exploded view, and (**D**) electrochemical diagram of the setup used for the experiments. The bioelectrode (working electrode, WE) was clamped using two PTFE disks which also held the platinum wire (counter electrode, CE) and the Ag/AgCl reference electrode (RE). The stainless-steel washer ensured electrical connection between the bioelectrode and the titanium wire.

A three-electrode electrochemical setup was used for the analysis. The bioelectrode was the working electrode, a platinum wire (ø = 0.10 mm, Advent Research Materials Ltd.) was used as a counter electrode, and an Ag/AgCl electrode was used as the reference electrode. The biosensor was clamped together, with a stainless-steel washer between two PTFE disks (Figure 2C). The CE and the reference electrode (RE) were held by the top part of the clamp. The experiments were performed using a MultiEmStat 4-channel potentiostat (PalmSens, Houten, the Netherlands) controlled by MultiTrace software. The electrochemical analyses were conducted using BG11 medium as an electrolyte at room temperature (22 ± 2 °C) to maintain an optimal environment for the bacterial cells. A white LED lamp (4W, 3000K; Verbatim) was used during the tests to provide illumination, at a distance of 20 cm resulting in the anodic surface being irradiated with ~450 µE m^−2^ s^−1^. Chronoamperometry was performed at +0.4 V vs.Ag/AgCl, and light was switched on and off in order to assess the photocurrent production of the electrode. During the inhibition experiments, herbicide solutions were directly injected in the electrolyte.

### 2.5. Chlorophyll Determination

Chlorophyll content of the biofilm present on the bioelectrodes for different lengths of time was determined by spectrophotometric measurement. Electrodes prepared as described above were kept at room temperature in the BG11 medium. Chlorophyll was extracted in 99.8% (v/v) methanol at 4 °C in the absence of illumination for 15 min under agitation. The content of chlorophyll *a* was calculated according to Porra et al. [52].

### 2.6. Biosensor Storage

In order to test their durability, several bioelectrodes prepared as previously described were placed in Petri dishes containing a sponge cloth (0.5 cm thickness, Houseproud) on the bottom **(**Figure S3). The sponge was moistened with BG11 medium to maintain the humidity inside the plates. The Petri dish was sealed with Parafilm^®^ and stored in a fridge at 4 °C.

## 3. Results

### 3.1. Biosensor Design and Photocurrent Production

**Figure 3:**
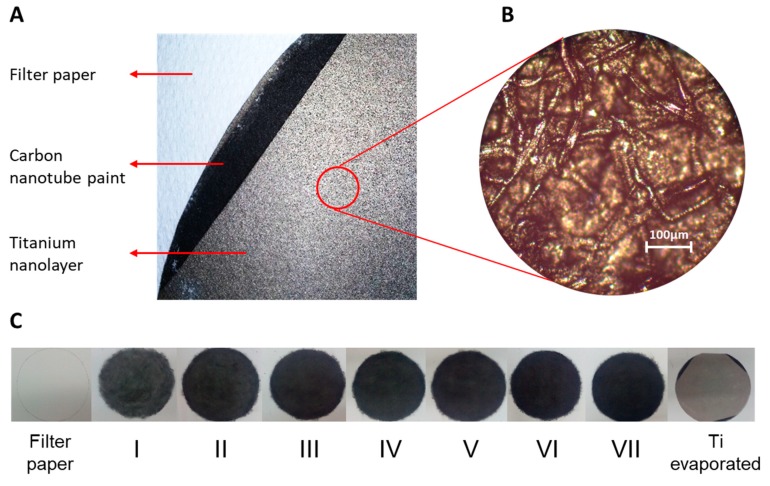
(**A**) Picture of the electrode surface, (**B**) image of the surface obtained with optic microscope, and (**C**) electrode construction. Filter paper was coated with seven layers of carbon nanotube paint and a topmost layer of titanium was added by evaporation. The diameter of the electrode, as shown in panel C, was 210 mm.

The novel electrochemical biosensor described here is shown in Figure 2. The system includes an anode made by filter paper coated with carbon nanotubes and a titanium nanolayer (Figure 3). The roughness of the filter paper provides a suitable surface for the cyanobacterial cells to adhere to. The anode is clamped between two Teflon disks. The electrochemical setup is completed with a counter electrode made by Pt wire and an Ag/AgCl reference electrode located on the top Teflon disk (Figure 2B,C). Cyanobacterial cells were allowed to sediment spontaneously on the anodic surface. More details on the fabrication of the anode are given in the Material and Methods section.

The novel electrochemical biosensor was tested using chronoamperometry, by applying +0.4 V bias potential vs. Ag/AgCl. No artificial redox mediator was added during the electrochemical analysis. The anodic surface was irradiated with white LED light at ~450 µE m^−2^ s^−1^.

**Figure 4:**
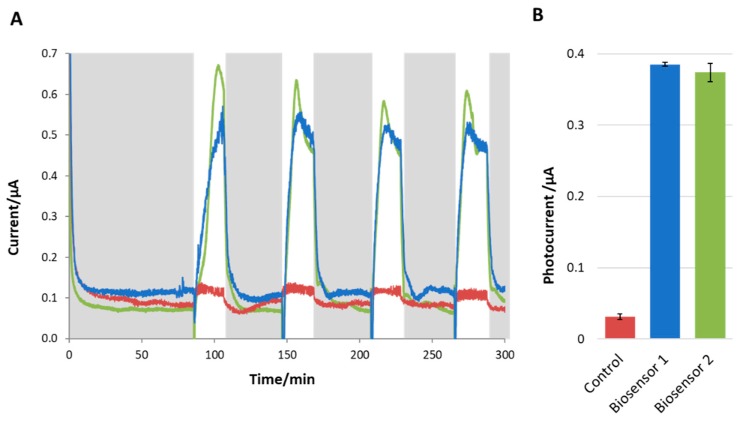
(**A**) The current output response to light and dark conditions for the biosensor developed here is shown in duplicate (blue and green lines). The abiotic control is shown on the same graph (red line). The periods of darkness are indicated with grey backgrounds, whereas the white background indicates illumination. The biosensors showed a significant and consistent response to light (i.e., increase) and dark (i.e., decrease) conditions. Photocurrent is calculated as the difference between the stable current under illumination and the background current. Only a very small change in current on illumination was observed in the control, where the biological material was not present. (**B**) Histogram of the photocurrent produced by the biosensors (replicate 1 and 2) and the control. The error bars represent the standard deviation between the different illumination cycles of the same biosensor.

As soon as the light was switched on (Figure 4A), a rapid increase in current (~0.4 µA) was observed for the biosensor operated with cyanobacterial cells. Then, when the dark condition was restored, the current returned to its preillumination level. This basal level was termed the background current. The photocurrent is defined as the difference between the current under illumination and the background current in the dark.

The photocurrent calculated during several cycles of illumination for the electrochemical biosensors operated with cyanobacterial cells was found to be stable over time and significantly higher than in the abiotic control (Figure 4B, *t*-test *p* = 0.01). Only a relatively small photocurrent (~0.03 µA) was recorded in the abiotic controls.

### 3.2. Detection of Herbicides

In order to evaluate the sensing capabilities of this device, the effect of the following three commonly used herbicides was tested: diuron, atrazine, and paraquat. In separate experiments the selected herbicides were injected into the cyanobacterial medium while performing chronoamperometry under cycles of dark and light. The percentage of inhibition was calculated by Equation (1).

(1) $$\mathrm{Phot}.\;\mathrm{Inibition}\;\left(\%\right)=\left(1-\frac{\mathrm{Phafter}}{\mathrm{Phbefore}}\right)*100$$

where Phafter is the photocurrent measured after inhibition and Phbefore is the photocurrent measured before treatment with the inhibitor, as shown in Figure S1.

**Figure 5:**
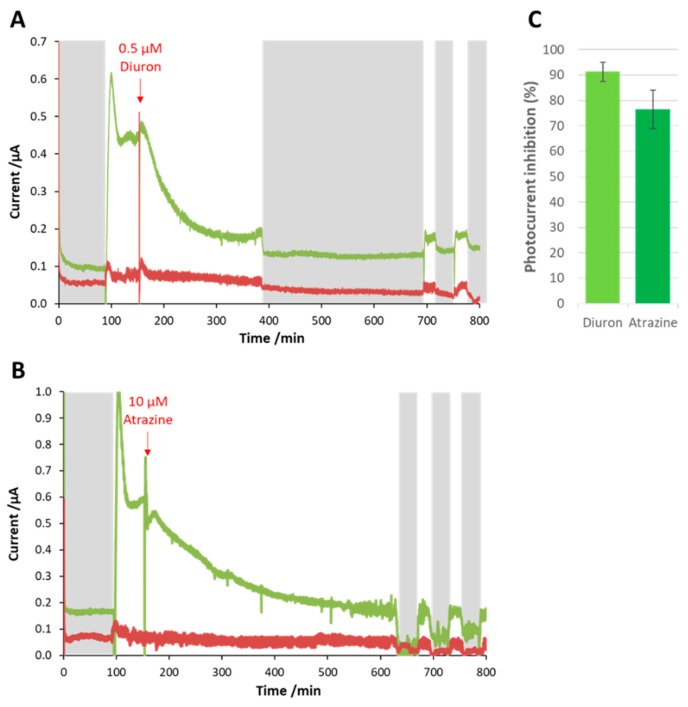
Effect of diuron (**A**) and atrazine (**B**) on the photocurrent of the biosensor (green lines). The periods of darkness are represented with grey backgrounds. Under illumination, the current decreased as soon as the herbicide was injected. For atrazine the inhibition process was slower as compared with diuron even though the concentration was more than an order of magnitude higher. The currents measured for the abiotic controls are shown with red lines. (**C**) Shows the percentage inhibition of photocurrent in the two cases. The error bars represent the standard deviation for three different bioelectrodes.

Both diuron (Figure 5A) and atrazine (Figure 5B) caused an immediate drop in the photocurrent, which continued to decrease gradually until it reached a constant level. The experiments were conducted in triplicate. The plots of the three replicates and relevant negative control can be found in the Supporting Information (Figures S2 and S3). Even though the concentration of diuron (0.5 µM) was more than one order of magnitude lower than that used for atrazine (10 µM), diuron caused a faster inhibition. Moreover, the inhibitory effect was significantly stronger in the case of diuron; atrazine was able to reduce the photocurrent by 76% ± 7%, versus 91% ± 4% reduction with diuron (Figure S4 and Table S1).

**Figure 6:**
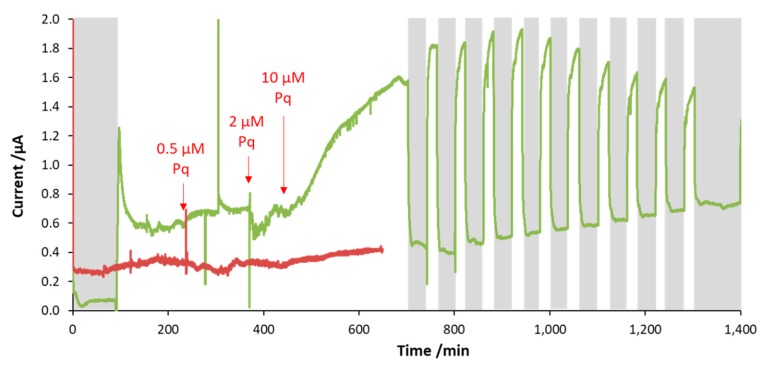
Effect of paraquat on the photocurrent (green line); when a small amount of the compound was injected (final concentration 0.5 µM) an increase in photocurrent was seen. For higher concentrations the photocurrent increased further and was more than doubled at 10 µM atrazine (final concentration) as compared with that in the absence of atrazine. However, after a few cycles of illumination the photocurrent decreased again to its initial level, while the dark current remained high. The current response of the abiotic control is shown with a red line.

In contrast, injection of paraquat resulted in an increase in the photocurrent of 203% ± 3% (Figure 6), for a few hours. Then, the photocurrent started to decrease. In addition, the background dark current showed a modest increase over time. This may indicate that paraquat was able to shuttle electrons originating from metabolic processes other than photosynthesis.

### 3.3. Bioelectrode Storage and Stability

**Figure 7:**
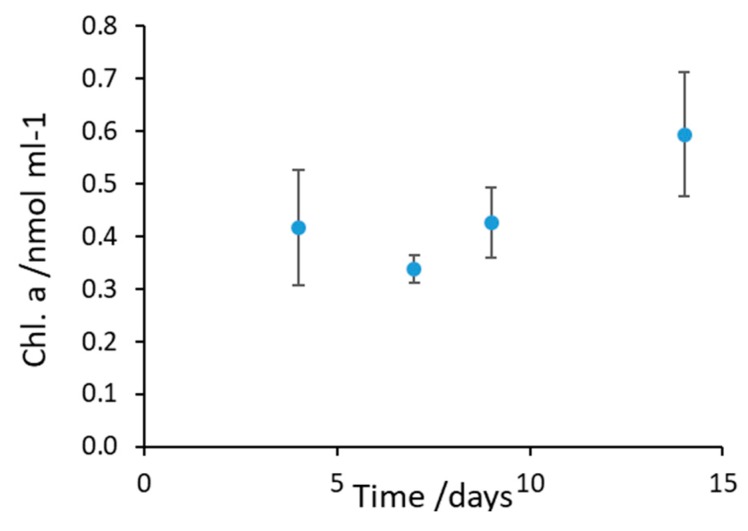
Stability of the cyanobacterial biofilm over time: chlorophyll-a content of the biofilm on the electrode surface measured over a period of 14 days.

The durability of the biosensor under storage for up to 22 days was investigated. Several bioelectrodes (i.e., anodes) were placed in Petri dishes containing a wet sponge to maintain humidity, as shown in Figure S5, and stored in a fridge at 4 °C. The viability and stability of the cyanobacterial biofilm on the electrode surface stored at 4 °C was tested by measuring the amount of chlorophyll-a [42] extracted from colonized electrodes at day 4, 8, 10, and 14 (Figure 7) [52]. The chlorophyll concentration remained substantially unchanged (slope of chlorophyll content against time was not significantly different from zero for Anova, *p* = 0.05). This result indicates that the biofilm on the anodic surface is composed of viable cells. If cells were unviable, the chlorophyll would be expected to degrade over the experimental time studied (14 days).

**Figure 8:**
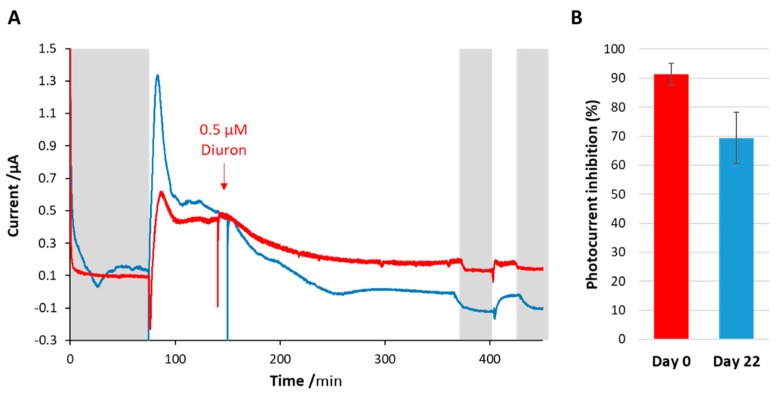
(**A**) After being stored in the fridge for 22 days (blue line), the biosensor showed similar performances in terms of photocurrent production and diuron detection as compared with a fresh biosensor on day zero (red line). (**B**) Shows the average photocurrent inhibition caused by diuron obtained with three different bioelectrodes for day zero and three different ones for day 22. The error bars represent the standard deviation of the three replicates.

Figure 8 shows a test conducted with a bioelectrode that was stored in this way for 22 days. The performance of this bioelectrode in terms of photocurrent and sensitivity towards diuron was slightly lower than for a freshly prepared biosensor. Indeed, only a loss of performance of ~20% was obtained, calculated as the difference in percentage inhibition of photocurrent between the two cases.

## 4. Discussion

When compared with other whole cell photosynthetic-based biosensors [34,37,40], the anode colonized by cyanobacteria displayed comparable stability in storage to the cases described in the literature (Table 1). However, the whole cell photosynthetic-based biosensors previously described were mainly based on monitoring the oxygen produced by photosynthesis and not on the direct electrochemical measurement of the photocurrent. Because the concentration of dissolved oxygen can be affected by other environmental factors (e.g., temperature), the potential for such devices to be used in the field might be limited. Recently, Chouler et al., used a MFC-based sensor to detect atrazine in the absence of photosynthetic bacteria [53]. However, the current output of MFCs is strongly influenced by the concentration of organic substrates fed to the anodic biofilm, which can mask or even overwhelm the response to the target toxicant [54]. 

The biosensor developed and tested in this study was able to detect the presence or absence of herbicide concentrations in the order of micromolars. When compared with the benchmark for acute exposure of fish set by the Environmental Protection Agency of the United States (US EPA) [55], the concentrations detected are below the threshold for acute exposure of fish (Table 2). Therefore, the sensor could be applied to environmental monitoring. For future development of commercial devices based on this proof of concept, further studies should test the effect of different herbicide concentrations on the sensor response in order to obtain calibration curves for the herbicide concentration versus photocurrent.

Although atrazine and diuron have the same mechanism of photocurrent inhibition, they showed different impacts on the photocurrent. Both compounds bind the QB pocket in PSII (Figure 1), thus interrupting the electron transport chain [56,57]. Nevertheless, atrazine caused a much slower inhibition as compared with diuron, even at 10-fold higher concentrations. In addition, the level of photocurrent inhibition caused by diuron was found to be higher than the inhibition caused by atrazine at the concentrations used (*t*-test, *p* = 0.05). These observations indicate that the biosensor is able to differentiate between the two substances to a certain extent. Indeed, diuron was previously reported as a stronger photosynthesis inhibitor than atrazine in terms of half maximal inhibitory concentration IC_50_ [41].

Our biosensor does not offer, at the moment, an absolute specificity for herbicides inhibiting photosynthesis. Other cytotoxic compounds (e.g., formaldehyde, heavy metals, other kinds of herbicides, etc.) could also cause photocurrent decrease [58]. However, the study of the profile of current output (i.e., chronoamperometry) may offer a way of discerning between different cytotoxic compounds, as demonstrated here in the case of atrazine and diuron. Moreover, the sensitivity towards a range of cytotoxic substance may be an advantage, as the sensor may be able to provide a warning signal for the presence of various harmful compounds in the environment.

The response towards paraquat was completely different, as it temporarily enhanced the photocurrent. This is probably due to its ability to shuttle electrons between bacterial cells and the electrode. This compound is a well-known redox mediator, and for this reason it is used in microbial electrochemical systems under anoxic conditions [59,60]. However, the fact that the enhancement was only short term might be due to paraquat’s cytotoxicity caused by the formation of peroxy radicals.

Electron transfer between *Synechocystis* and the electrode was achieved without adding any exogenous electron carrier. Under this condition, a net photocurrent production was obtained, with good reproducibility. This aspect gives several advantages to the biosensor. Redox mediators are often toxic and can increase the operational cost and complexity. Because of their cytotoxicity, the enhancement of the photocurrent with redox mediators is typically temporary [45]. In addition, quinone-based mediators may undergo photodegradation when dissolved in an aqueous media [61], and thus their activity decreases over time.

Our data suggest that, in absence of any exogenous electron carries, the biosensor could remain functional after over 20 days of storage at low temperature, with only a small loss in performance (~20%). The retention of activity was probably achieved thanks to the capacity of the cyanobacterial biofilm to regenerate or stay dormant. In the context of field applications, such as monitoring of water bodies or agricultural areas, the ability of this biosensor to recover after a cold season could be a key advantage.

However, further investigations should extend the present study, especially focusing on the durability of the cyanobacterial anodic biofilm over subsequent cycles of herbicide inhibition and recovery. In addition, native cyanobacteria kept in the dark, tend to lose their photosynthetic activity [62]. The lifetime of the photoanodic population might represent a limiting factor for commercial application of these kind of devices. Furthermore, the behavior of the biosensor at different temperatures should be analyzed, as this factor can affect the metabolism of the biological receptor.

## 5. Conclusions

A mediatorless electrochemical biosensor operating with wild type *Synechocystis* PCC6803 was developed and tested. The biosensor was able to detect the presence of diuron and atrazine at the micromolar level, which are concentrations relevant to environmental analysis. Additionally, we showed that our cyanobacteria-colonized electrode could successfully recover after more than 20 days of low-temperature conditions. This concept opens up potential applications in the field of environmental monitoring. However, further research is needed to assess the stability of the photosynthetic anodic biofilm over time and to enhance the sensitivity to different concentrations. In addition, further optimization of the storage conditions (e.g., temperature, light intensity, and moisture) is needed in order to improve the storage stability of the bioreceptor.

## Figures and Tables

**Table 1:** Comparison of the storage stability of amperometric photobiosensors found in literature and the present sensor.

Bioreceptor	Storage Conditions	Storage Time (days)	Loss of Sensitivity (%)	Reference
*Chlorella vulgaris*	4 °C in CaCl_2_ solution	30	20	Shitanda et al., 2009 [37]
*Chlorella vulgaris*	4 °C in Tris-HCl + MgCl_2_	10	45	Ionescu et al., 2006 [34]
*Chlamydomonas reinhardtii*	25 °C in storage buffer, light	25	40	Husu et al., 2013 [40]
*Synechocystis 6803 wt*.	4 °C in BG11 medium, dark	22	22	Present study

**Table 2:** Decrease in photocurrent upon treatment with the tested herbicide expressed as a percentage (average for three replicates), concentrations used for the experiments, and threshold of acute exposure for fish set by the Environmental Protection Agency of the United States (EPA Aquatic life benchmarks [55]). The biosensor could detect the presence of the selected compounds before it reached the threshold in all three cases.

	Photocurrent Variation (%)	Concentration Used (μM)	Acute Exposure for Fish (μM)
**Atrazine**	−76 ± 7	10.7	12.3
**Diuron**	−91 ± 4	0.5	0.9
**Paraquat**	+203 ± 3	0.7	23.3

## Supplementary Materials

The following are available online at https://www.mdpi.com/2076-2607/7/12/630/s1.

## Abbreviations

CN	carbon nanotubes
WE	working electrode
CE	counter electrode
RE	reference electrode
ITO	indium tin oxide
Pq	paraquat
PS I	photosystem I
PS II	photosystem II
