# Supplementary material for: A Storable Mediatorless Electrochemical Biosensor for Herbicide Detection
Source: Microorganisms. 2019 Nov 29;7(12):630. doi: 10.3390/microorganisms7120630 (PMC6956157; doi:10.3390/microorganisms7120630)
Supplement: Supplementary file 1 [file microorganisms-07-00630-s001.pdf]

Supporting information

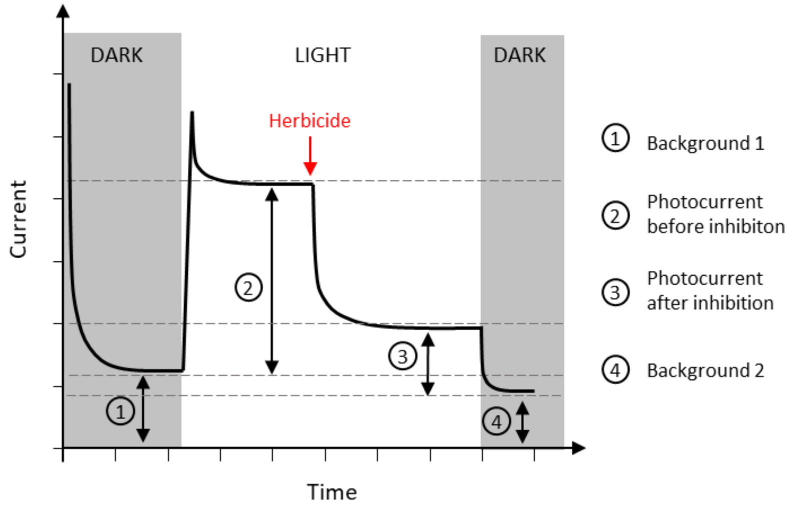

Figure S1. Representative chronoamperometry with annotated phases.

A

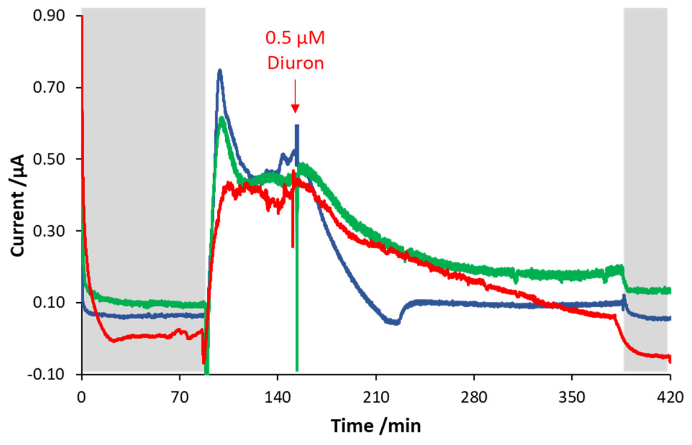

B

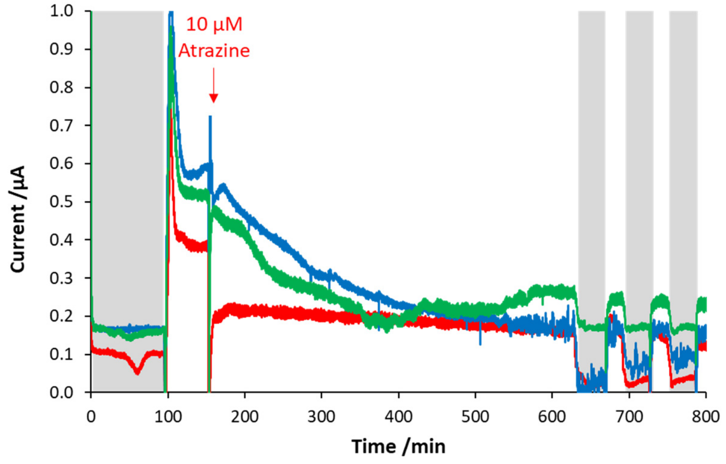

C

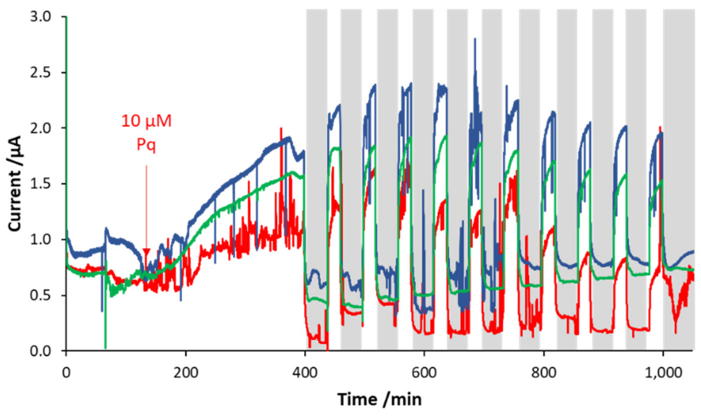

Figure S2. Triplicates of the chronoamperometric detection of herbicides for A) diuron, B) atrazine and C) paraquat. The grey background indicates the periods of darkness. The applied potential was +0.4V vs. Ag/AgCl.

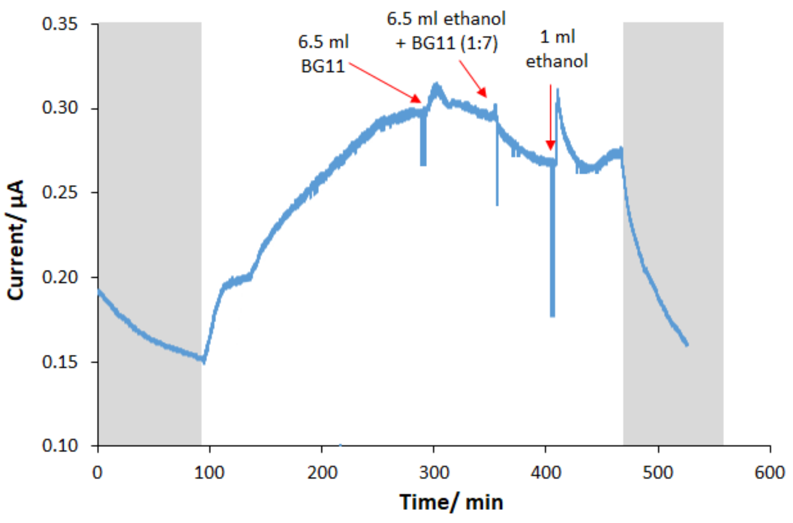

Figure S3. Chronoamperometric detection performed with the biosensor where the solvent used for paraquat (6.5mL BG11), atrazine (6.5mL ethanol:BG11 1:7) and diuron (1 mL ethanol) in absence of herbicide were added in the electrochemical cell operated with biotic anode and containing 170ml of BG11. The grey background indicates the periods of darkness. The applied potential was +0.4V vs. Ag/AgCl.

Table S1. Photocurrent production before and after the injection of the tested herbicides. The photocurrent is calculated as the difference between the current obtained during illumination and the current obtained in darkness.

	DIURON		ATRAZINE		PARAQUAT	
	**Average**	**St. Dev.**	**Average**	**St. Dev.**	**Average**	**St. Dev.**
**Photocurrent (µA)**	0.397	0.051	0.353	0.067	0.487	0.038
**Photocurrent + Herbicide (µA)**	0.033	0.012	0.080	0.010	1.477	0.124
**Variation %**	-91	4	-76	7	203	3

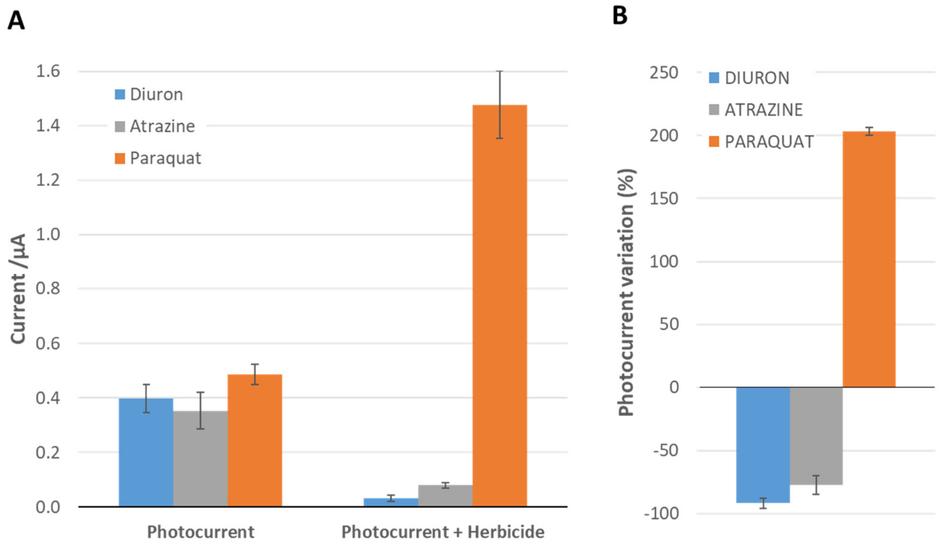

Figure S4. (A) photocurrent production before and after the injection of the tested herbicides. (B) Variation of photocurrent after the injection of herbicide expressed as percentage.

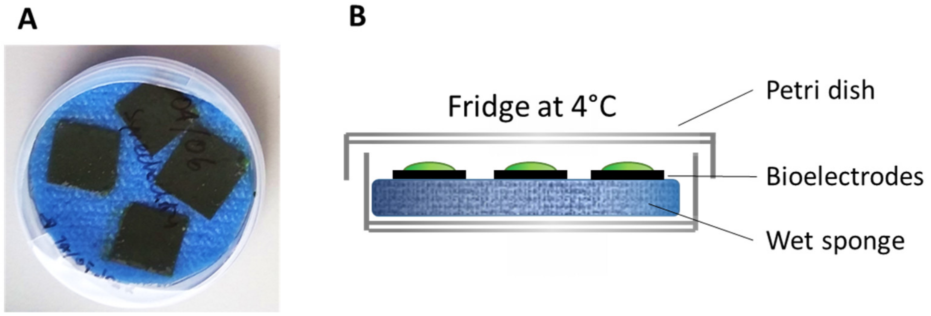

Figure S5. Setup used for storing the bioelectrodes in the fridge: A) picture of the Petri dish containing the bioelectrodes on top of the sponge, B) schematic representation of the setup
